# Ferroptosis and the bidirectional regulatory factor p53

**DOI:** 10.1038/s41420-023-01517-8

**Published:** 2023-06-29

**Authors:** Ren Xu, Wanning Wang, Wenlong Zhang

**Affiliations:** 1 Pulmonary and Critical Care Medicine Department, First Hospital of Jiliwn University, 130021 Changchun, China; 2 Nephrology Department, First Hospital of Jilin University, 130021 Changchun, China; 3 Department of Hematology and Oncology, China-Japan Union Hospital of Jilin University, 130033 Changchun, China

**Keywords:** Cell death, Cancer

## Abstract

Ferroptosis is a type of regulated cell death characterized by iron-mediated lipid peroxidation, in contrast with apoptosis, autophagy, and necrosis. It can be triggered by many pathological processes, including cellular metabolism, tumors, neurodegenerative diseases, cardiovascular diseases, and ischemia–reperfusion injuries. In recent years, ferroptosis has been discovered to be associated with p53. P53 is a tumor suppressor protein with multiple and powerful functions in cell cycle arrest, senescence, cell death, repair of DNA damage, and mitophagy. Emerging evidence shows that ferroptosis plays a crucial role in tumor suppression by p53. P53 functions as a key bidirectional regulator of ferroptosis by adjusting metabolism of iron, lipids, glutathione peroxidase 4, reactive oxygen species, and amino acids via a canonical pathway. In addition, a noncanonical pathway of p53 that regulates ferroptosis has been discovered in recent years. The specific details require to be further clarified. These mechanisms provide new ideas for clinical applications, and translational studies of ferroptosis have been performed to treat various diseases.

## Facts

- P53 plays a crucial role in ferroptosis. It targets the key proteins and enzymes involved in ferroptosis such as SLC25A28, FDXR, SAT1-ALOX15, GLS2, SLC7A11, DPP4, p21, ALOX12, and iPLA2β. P53 also regulates noncoding RNAs to promote ferroptosis.
- Regulation of ferroptosis by p53 is complex, bidirectional, and context dependent. P53 promotes ferroptosis to help the body eliminate tumor cells and abnormal cells. P53 can reduce the sensitivity of cells to ferroptosis and promote normal cell survival.
- Translational research of p53 and ferroptosis demonstrates potential clinical prospects for tumors and ischemia–reperfusion injury.

## Introduction

A form of iron-dependent cell death that cannot be hindered by genetic or chemical inhibitors of necroptosis or apoptosis was first reported a decade ago and named ferroptosis [1]. In subsequent research, researchers found that ferroptosis differs from apoptosis and necrosis in terms of genetics, biochemistry, and morphology [2]. It is characterized by lethal accumulation of iron and lipid hydroperoxides, and subsequent plasma membrane rupture and cell death [3]. There are a wide range of pathological conditions and human diseases associated with ferroptosis, including organ injury [4–6], organ fibrosis [7], tumors [8–12], neurodegenerative diseases [13], cardiovascular diseases [14], and gynecological diseases [15]. The complex mechanisms and cellular metabolic processes involved in ferroptosis are gradually being elucidated.

The tumor suppressor protein p53 was first discovered in the 1970s [16, 17]. P53 is at the center of a signaling network controlling cell proliferation and death, and is the guardian of genome integrity. The concentration of p53 protein in normal cells is very low, and p53 is activated when DNA damage, nutritional deficiency, hypoxia, or oxidative stress occur. P53 is a meaningful regulatory factor in ferroptosis that promotes or inhibits ferroptosis through canonical and noncanonical pathways. As a vital regulatory factor, p53 and its pathways provide potential targets for clinical treatment and novel interventions for many diseases.

## Process of ferroptosis

Cells undergoing ferroptosis have unique morphological features, including lessened mitochondrial size, increased mitochondrial membrane density, reduced/absent mitochondrial cristae, ruptured mitochondrial outer membranes, and a normally sized nucleus but with no concentration of chromatin [2]. The process of ferroptosis involves iron, lipid, and reactive oxygen species (ROS) metabolism and antioxidant systems.

### Abnormal metabolism and subsequent accumulation of iron

**Fig. 1:**
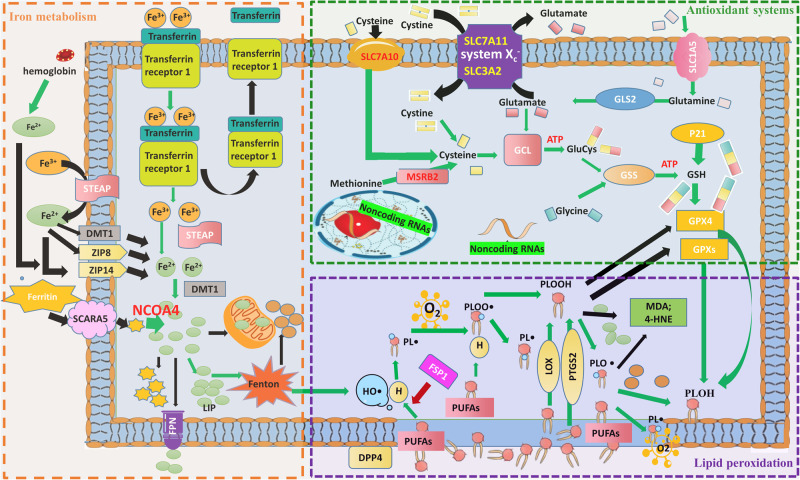
The process of ferroptosis and the involvement of iron, lipid, and GSH metabolism. Intracellular excess Fe^2+^ enters the labile iron pool, causing the Fenton reaction, in which Fe^2+^ is oxidized to Fe^3+^, while the electron is transferred to H_2_O_2_ to form HO•. HO• initiates the oxidation of PUFAs of membrane PLs. The PUFA of a PL donates a hydrogen atom to HO•, leading to formation of a carbon-centered PL• that further reacts with intracellular molecular O_2_ to form a PL peroxyl radical (PLOO•). Next, from another PUFA moiety of a PL, PLOO• abstracts a hydrogen atom and is subsequently converted to PL hydroperoxide (PLOOH) accompanied by formation of a new PL•. PLOOH is cleaved in the presence of Fe^2+^ to form the PL alkoxyl radical (PLO•), which reacts with the PUFA of another PL to form PL alcohol (PLOH) and a new PL•, followed by another lipid radical chain reaction. There are three types of lipid oxidation enzymes: cyclooxygenase (COX), cytochrome P450 (CYP), and lipoxygenase (LOX). They initiate the enzymatic lipid peroxidation process. Lipid peroxides including PL free radicals can be eliminated by GPXs, MDA, or 4-HNE, but excess PL free radicals cause the unbounded peroxidation of PUFAs of membrane PLs, which eventually leads to destruction of the cell membrane. Intracellular Fe^2+^ and GPX4 are generated via iron and GSH metabolism, respectively. The red arrow represents the inhibitory effect, and the green arrow represents the promoting effect. 4-HNE 4-hydroxynonenal, DMT1 divalent metal transporter 1, DPP4 dipeptidyl peptidase-4, FPN ferroportin (also known as SLC40A1), GCL glutamate-cysteine ligase, GLS2 glutaminase 2, GPX4 glutathione peroxidase 4, GSH glutathione, GSS glutathione synthetase, HO• hydroxyl radical, LIP labile iron pool, LOX lipoxygenase, MDA malondialdehyde, MSRB2 methionine-R-sulfide reductase B2, NRF2 nuclear factor erythroid 2-related factor 2, p21 cyclin-dependent kinase inhibitor 1A, i.e., CDKN1A, PL phospholipid, PL• PL radical species, PLO• PL alkoxyl radical, PLOH PL alcohol, PLOO• PL peroxyl radical, PLOOH, PL hydroperoxide, PTGS2 (encodes cyclooxygenase-2, which is an enzyme that acts both as a peroxidase and a dioxygenase, and catalyzes lipid oxidation), PUFA polyunsaturated fatty acids, SCARA5 scavenger receptor class A member 5, SLC1A5 solute carrier family member 1 member A5, SLC7A11 solute carrier family 7 member A11, STEAP six transmembrane epithelial antigen of prostate, TFR1 transferrin receptor 1, ZIP8 Zrt- and Irt-like protein 8.

Iron is a material nutrient and has important biological functions, such as catalyzing various biochemical reactions by accepting and donating electrons [18]. Metabolic processing of iron includes its import, utilization, storage, and excretion from cells. Ferroptosis is triggered by an overload of intracellular iron, which is associated with abnormal iron metabolism (Fig. 1).

Cells import iron in four ways [18]. The most critical path is mediated by transferrin and transferrin receptor 1 (TFR1). Transferrin is a type of globulin that can chelate two Fe^3+^ ions and bind to TFR1. Then the Fe^3+^-transferrin-TFR1 complex is internalized via endocytosis and release Fe^3+^ which is reduced to Fe^2+^ [19]. Fe^2+^ subsequently enters the cytoplasm labile iron pool (LIP) via the action of divalent metal transporter 1 (DMT1). Furthermore, there are several types of ferri-reductases in serum, such as six transmembrane epithelial antigen of prostate (STEAP), ferric chelate reductase 1, and cytochrome B reductase 1, which can convert Fe^3+^ to Fe^2+^. In addition, Fe^2+^ enters cells through membrane transporters (such as ZIP8, ZIP14, and DMT1). Monocytes and macrophages can take up Fe^2+^ from porphyrin contained in hemoglobin [20]. In embryos and kidneys, ferritin enters cells through scavenger receptor class A member 5 [21].

Intracellular Fe^2+^ is transported into mitochondria to assist with cellular respiration and synthesis of Fe-S clusters and heme by mitoferrin 1 and 2 [22]. Some Fe^2+^ is stored in cells in the form of the LIP or ferritin. Fe^2+^ in the LIP is unstable, and can regulate cellular iron homeostasis and induce ferroptosis. The ferritin protein sequesters iron and is involved in iron transport and cell proliferation, immunosuppression, and angiogenesis [23]. Ferritin releases free iron through the action of nuclear receptor coactivator 4 (NCOA4) in a process named ferritinophagy [24]. NCOA4 is responsible for recruiting ferritin heavy chain polypeptides into autophagosomes so that lysosomes can degrade ferritin, resulting in an increase of free iron [25]. An increase in iron content triggers NCOA4 to interact with HERC2, an E3 ubiquitin ligase, which promotes ubiquitination and degradation of NCOA4. Knockdown of NCOA4 or autophagy-related gene (ATG) can inhibit ferritin degradation and ferroptosis induced by erastin, while NCOA4 overexpression promote ferroptosis.

Iron export relies on ferroportin-1 (FPN, also known as SLC40A1). FPN cooperates with ceruloplasmin or hephaestin, and transports Fe^2+^ out of cells [26]. It is the unique iron efflux pump, and its expression is dramatically suppressed in many types of tumors [27]. Compared to non-tumor cells, tumor cells have a high iron requirement and dependency, and therefore are more likely to undergo ferroptosis in response to ferroptosis-inducing drugs, and this is a promising novel way to kill therapy-resistant cancers [28].

Iron is utilized, stored, and exported in a balanced manner. Iron accumulation caused by abnormal metabolism is the basis of ferroptosis. Various iron chelators such as ferrostatin-1 [29] and liproxstatin-1 can inhibit ferroptosis. The process after accumulation of iron includes lipid oxidation and inactivation of antioxidant systems.

### Intracellular accumulation of iron-induced ROS

When too much Fe^2+^ enters the cell, the utilization or exportation of Fe^2+^ is reduced and the intracellular Fe^2+^ level increases. Excess Fe^2+^ initiates the Fenton reaction. The process of the Fenton reaction is as follows: Fe^2+^ is oxidized to Fe^3+^, while the electron is transferred to hydrogen peroxide (H_2_O_2_) to form the hydroxyl radical (HO•), which is the most chemically active ROS [30]. In addition to HO•, another two ROS molecules, superoxide (O_2_•^-^) and H_2_O_2_, are involved in iron redox reactions. ROS function as driver of ferroptosis by triggering lipid peroxidation, and they can mediate autophagy to induce ferroptosis by degrading ferritin and increasing TFR1 expression upon exposure to erastin [31].

### Lipid metabolism and peroxidation

Lipid peroxidation induced by iron accumulation plays a central role in ferroptosis. Phospholipids (PLs), which are divided into sphingomyelin and glycerophospholipids, are the basic components of cellular membranes. The major fatty acids in sphingomyelin are saturated fatty acids or monounsaturated fatty acids, which cannot induce ferroptosis. Glycerophospholipids contain a saturated fatty acid at C-1, an unsaturated fatty acid (PUFA) [arachidonic acid (AA)] at C-2, and a phosphatidic ester group at C-3 of the glycerol backbone. Peroxidation of PUFAs at C-2 can drive ferroptosis. De novo synthesized fatty acids must be incorporated into PLs by acyl-CoA synthetases (ACSs). Phosphatidylethanolamines containing AA or adrenic acid (AdA) are key PLs that can be oxidized and drive ferroptosis. ACS long-chain family member 4 (ACSL4) first converts AA to acylated AA, and lysophosphatidylcholine acyltransferase 3 catalyzes the incorporation of acylated AA into the PL.

A study [32] confirmed that the expression of ACSL4 in a cell subgroup of triple negative breast cancer is related to its sensitivity to ferroptosis inducers. A similar correlation was observed in drug-resistant mesenchymal carcinoma cells and renal carcinoma cells. Inhibition of ACSL4 expression may be the main mechanism underlying desensitization of cells to ferroptosis. On the contrary, elevation of the expression or activity of ACSL4 may promote ferroptosis under various pathophysiological conditions, such as ischemia–reperfusion injury, and in response to radiation.

ROS (mainly HO•) produced by the Fenton reaction initiate the nonenzymatic free radical chain oxidative reaction of PUFAs on cell membranes and thereby change these membranes to instigate ferroptosis [33]. The oxidized PUFAs on cellular membranes can disturb the assembly of these membranes; change the properties of lipid bilayers in terms of disrupted ion gradients, decreased fluidity, slower lateral diffusion, and increased permeability; influence lipid-lipid and lipid-protein interaction dynamics; destroy the physicochemical properties of membranes; influence the transport of nutrients; affect membrane-initiated signaling pathways; and impact metabolic processes, eventually leading to cell death [33].

Lipid peroxidation can be divided into spontaneous oxidation and enzymatic oxidation. Spontaneous oxidation of lipids is a chain reaction driven by free radicals (Fig. 1). The hydrogen of a PUFA is obtained by the hydroxyl group to form a carbon-centered lipid radical (L•). The rapid reaction of oxygen molecules with L• produces lipid peroxide groups (LOO•). Subsequently, LOO• obtains hydrogen from a nearby PUFA to form hydroperoxide (LOOH) and a new L•, and LOOH is converted to an alkoxy group (LO•) with the cooperation of Fe^2+^. These new L• and LO• groups react with an adjacent PUFA to cause another chain reaction. This spontaneous process is catalyzed by iron and oxygen. In addition, some lipoxygenases (LOX) can directly oxidize PUFAs and PUFA-containing lipids in biofilms, suggesting that LOX mediate ferroptosis [34]. In the process of enzyme-catalyzed lipid peroxidation, a PUFA is introduced into membrane PLs and neutral lipids, and produces large amounts of lipid peroxidation products, 4-hydroxynonaenoic acid (4-HNE), malondialdehyde, PL LOOH (PLOOH), and various oxidized and modified proteins. Knockout of arachidonate 15-lipoxygenase (ALOX15) or application of the LOX inhibitor baicalein can protect mice from ischemic injury.

Ferroptosis suppressor protein 1 (FSP1) is a ferroptosis resistance factor that was identified in recent years [35]. It can prevent PL peroxidation, especially in the presence of both α-tocopherol and CoQ_10_. In the presence of FSP1, CoQ_10_ is reduced to CoQ_10_-H_2_ by NADPH to inhibit the propagation of PL peroxidation.

### Impairment of the system X_c_^-^-GSH-GPX4 axis promotes ferroptosis

To diminish oxidative damage, cells have many antioxidant systems. As the main detoxifying factor for lipid peroxides, glutathione peroxidase 4 (GPX4) converts active PLOOH to inactive PLOH, and thus inhibits lipid peroxidation. This is also an important regulatory target of the canonical ferroptosis pathway (Fig. 1) [36]. GPX4 is a selenocysteine-containing glutathione peroxidase. It can inhibit the hydrogen peroxide reaction of PLs and cholesterol, even when they are embedded in membranes and lipoproteins. GPX4 overexpression prevents ferroptosis induced by RSL3, while knockdown or inhibition of GPX4 promotes it [37].

GSH is a cofactor of the lipid peroxide reductase GPX4. The level of intracellular GSH is regulated by the cystine-glutamate antiporter (system X_c_^-^) [38]. SLC7A11 is a crucial component of system X_c_^-^, which is located on the cell surface and is responsible for the exchange of extracellular cystine and intracellular glutamate. Once cystine enters the cell, its disulfide bond breaks to form cysteine, which is one of three substrates for GSH synthesis. Thus, inhibition of SLC7A11 reduces the import of cystine and subsequently reduces synthesis of GSH, which greatly decreases the level of GSH in cells, and this is followed by lower elimination of lipid peroxides via GPX4 [2].

Beclin 1 is a key regulator of macroautophagy/autophagy. It can promote ferroptosis through inhibition of system X_c_^-^ [39]. Beclin 1 is phosphorylated by the AMP-activated protein kinase (AMPK) to initiate complex formation with SLC7A11 [39]. In beclin 1-overexpressing cells, the level of the beclin 1-SLC7A11 complex is increased, GSH depletion is serious, and the lipid oxidative stress reaction is aggravated, which promotes ferroptosis. On the contrary, after deletion of the gene encoding beclin 1, formation of the beclin 1-SLC7A11 complex is blocked, the level of the antioxidant GSH is increased, and cells are resistant to ferroptosis. Both vivo and vitro experiments found that the Tal-beclin 1 activating peptide can promote ferroptosis induced by erastin [40].

## P53 and ferroptosis

In recent years, a growing number of studies have confirmed that p53 is closely associated with metabolic pathways of ferroptosis and is a critical regulatory factor of ferroptosis [8]. Regulation of ferroptosis by p53 is complex, context-dependent, and tissue-specific, and can promote the occurrence of ferroptosis and also prevent it. When lipid peroxidation damage is mild and can be repaired, p53 helps cells to restrain ferroptosis. However, if the damage persists or is too severe, p53 will induce ferroptosis and damaged cells will be removed. Regulation of ferroptosis by p53 can be divided into the canonical and noncanonical pathways according to whether it depends on GPX4.

### The canonical pathway of p53 that regulates ferroptosis

**Fig. 2:**
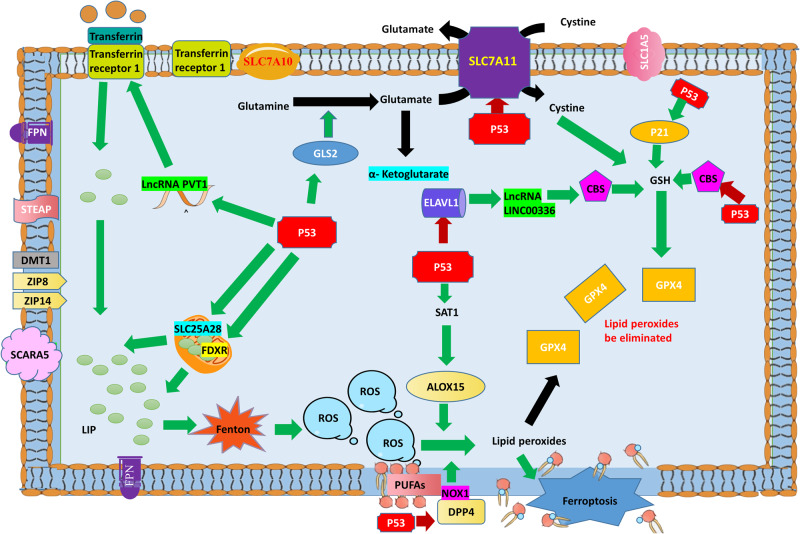
The canonical pathway of p53 that regulates ferroptosis. Iron metabolism: p53 promotes entry of Fe^2+^ into cells by regulating the lncRNA PVT1 to increase expression of TFR1, and promotes production of prototype iron by regulating SLC25A28 and FDXR, thus promoting ferroptosis. ROS metabolism: In the absence of amino acids, p53 promotes ROS production and lipid metabolism by promoting expression of GLS2. Lipid peroxidation: p53 increases the level of ALOX15 by promoting expression of SAT1, and then ALOX15 promotes lipid peroxidation to induce ferroptosis. P53 also inhibits DPP4-dependent lipid peroxidation to resist ferroptosis by transferring DPP4 to the nucleus. Elimination of lipid peroxides: p53 downregulates the level of CBS by inhibiting ELAVL1, or directly inhibits expression of CBS, thereby limiting GSH production. P53 also inhibits synthesis of GSH by inhibiting SLC7A11. Thus, p53 indirectly inhibits the activity of GPX4, reducing intracellular elimination of lipid peroxides and finally promoting ferroptosis. P53 also induces expression of p21, and then production of GSH increases, which increases GPX4 activity and the ability of cells to eliminate lipid peroxides. The green arrow indicates the stimulatory effect and the red arrow indicates the inhibitory effect.

The canonical pathway of p53 that regulates ferroptosis involves metabolism of iron, lipids, amino acids, and ROS (Fig. 2). The core factor is GPX4.

#### P53 and iron metabolism

P53 regulates ferroptosis by affecting iron metabolism and ferredoxin reductase (FDXR) levels [41]. There are two targets of p53 in mitochondria: solute carrier family 25 member 28 (SLC25A28) and FDXR. In hepatic stellate cells, p53 enhances the activity of SLC25A28, and thereby causes abnormal assembly of redox-active iron and augments ferroptosis [42]. FDXR is an enzyme in mitochondria that can transfer electrons from NADPH to cytochrome P450. Under the action of RSL3 or erastin, p53 induces transcriptional expression of FDXR to promote ferroptosis [41]. The exact effect and mechanism remain to be clarified. The p53-FDXR loop maintains mitochondrial iron homeostasis and is critical for tumor suppression. This adaptive regulatory mechanism requires further study.

#### P53 and ALOX15

P53 can regulate the p53-spermidine/spermine N1-acetyltransferase 1 (SAT1)-ALOX15 metabolic pathway to promote ferroptosis [43]. SAT1 is a rate-limiting enzyme that regulates conversion of spermidine and spermine to putrescine during polyamine catabolism. SAT1 is also a direct target of p53. Under oxidative stress, upon treatment with the DNA-damaging agent doxorubicin, or induction by Nutlin-3 (a small molecule MDM2 inhibitor), p53 targets SAT1 and promotes its expression. This increases the level of ALOX15, which oxygenates PUFAs to promote ferroptosis without affecting SLC7A11 or GPX4 [43].

#### P53 and glutamine metabolism

Glutamine metabolism is another pathway involved in the regulation of ferroptosis. Glutaminase-2 (GLS2) is a mitochondrial enzyme that catalyzes the first step of glutamine catabolism to glutamate. GLS2 is also a direct transcriptional target of p53 to positively regulate ferroptosis. In the absence of amino acids or even entire or partial depletion of cystine, glutamine can induce ferroptosis in a serum-dependent manner [44]. P53 binds to the response element in the *GLS2* and induces its transcription. Thereafter, GLS2 converts glutamine into glutamate and then into α-ketoglutarate, and the low level of GSH inhibits GPX4, which promotes ferroptosis [45]. Jennis et al. found a p53 variant (called S47) in African-descent populations that has impaired GLS2 transactivation ability [46]. Tumor cells with S47 have a decreased level of GLS2 and are resistant to ferroptosis. Mice with S47 are susceptible to spontaneous cancers. These findings indicate that ferroptosis is crucial for the mechanism underlying p53 tumor inhibition by targeting GLS2.

#### P53 and system X_c_^-^/GPX4

As mentioned earlier, GPX4 is a selenocysteine-containing glutathione peroxidase that reduces lipid peroxides to the corresponding alcohols [47]. Regulation of GPX4 by p53 is bidirectional. P53 inhibits GPX4 by inhibiting the SLC7A11-GSH pathway or cystathionine-β-synthase (CBS), and then promotes ferroptosis. It also promotes production of GPX4 through the p21/GSH pathway to prevent ferroptosis.

Jiang et al. first reported that p53 transcriptionally inhibits SLC7A11 to sensitize cells to ferroptosis [8]. They identified that p53 directly targets the responsive element in the promoter of *SLC7A11* to inhibit its expression, and then a cascade reaction occurs. As uptake of extracellular cystine declines, GSH synthesis falls, GPX4 activity decreases, the level of lipid peroxides increases, and finally ferroptosis occurs. P53 also inhibits the serine trans-sulfuration pathway by repressing CBS. This limits GSH production and thereby indirectly inhibits the enzymatic activity of GPX4, eventually promoting ferroptosis [48, 49].

p53 can also promote ferroptosis induced by RSL3 or erastin through upregulation of *PTGS2*. *PTGS2* encodes cyclooxygenase-2, which is an enzyme that acts both as a peroxidase and a dioxygenase, and catalyzes lipid oxidation. A study by Yang et al. revealed that *PTGS2* is the most upregulated gene during erastin- or RSL3-induced ferroptosis [50].

#### P53 and noncoding RNAs

P53 also can induce ferroptosis by regulating noncoding RNAs. It regulates expression of numerous noncoding RNAs, including microRNAs (miRNAs) and long noncoding RNAs (lncRNAs) [51–53]. Meanwhile, p53 is also reciprocally regulated by various noncoding RNAs. The embryonic lethal vision-like protein 1 (ELAVL1)-LINC00336-miR-6852-CBS and PVT1-miR-214-TFR1 axes are well studied. ELAVL1 is an ELAV-like RNA-binding protein that binds to and stabilizes the lncRNA LINC00336 in lung cancer cells [49]. This stabilizes the miRNA miR-6852 and increases expression of CBS, making cells insensitive to ferroptosis. P53 promotes ferroptosis by binding to the promoter region of *ELAVL1* and inhibiting its expression. This is followed by destabilization of LINC00336 and release of miR-6852. As the level of CBS decreases, ferroptosis is promoted.

In another axis, the lncRNA PVT1 promotes ferroptosis by restraining the miR-214-mediated downregulation of TFR1. P53 can induce expression of PVT1 to promote ferroptosis in cancer and acute ischemic stroke-induced tissue injuries [54, 55].

#### P53 and dipeptidyl peptidase-4 (DPP4)

DPP4 is a ubiquitous enzyme located on the cell membrane or in a soluble form. It activates lipid peroxidation by interacting with NADPH oxidase 1 (NOX1), leading to ferroptosis [56]. In human colorectal carcinoma, p53 forms a complex with DPP4 and translocates it to the nucleus, and thus inhibits plasma membrane-associated DPP4-dependent lipid peroxidation to restrain ferroptosis [56].

### The noncanonical pathway of p53 that regulates ferroptosis

In the noncanonical pathway, p53 regulates ferroptosis through the arachidonate 12-lipoxygenase (ALOX12), arachidonate lipoxygenase 3 (ALOXE3), or iPLA2β pathway. These are also potential hot research topics.

#### P53 and ALOX12/ALOXE3

**Fig. 3:**
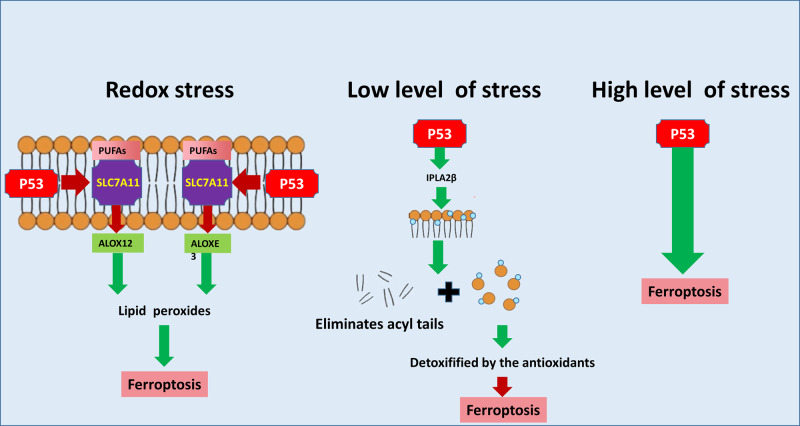
The noncanonical pathway of p53 that regulates ferroptosis. Following redox stress, p53 enhances the activities of ALOX12 and ALOXE3 by downregulating SLC7A11. Then, ALOX12 and ALOXE3 are separately released and subsequently oxidize membrane PUFAs, to produce lipid peroxides and initiate ferroptosis. When lipid peroxidation damage is low and can be repaired, p53 targets IPLA2β, which eliminates acyl tails from lipids and releases oxidized fatty acids. The latter are further detoxified by antioxidants in the cytoplasm, and thus ferroptosis is inhibited. When the damage persists or is too severe, p53 induces ferroptosis and thereby eliminates damaged cells. The green arrow indicates the stimulatory effect and the red arrow indicates the inhibitory effect.

ALOX12 and ALOXE3 belong to the lipoxygenase family, and are involved in the noncanonical pathway of p53-mediated ferroptosis regulation that is independent of GSH and GPX4. Under normal conditions, SLC7A11 binds to and sequesters ALOX12 from its substrate, PUFAs, including those esterified in membranes [57]. In response to redox stress, p53 enhances the activity of ALOX12 by downregulating SLC7A11 (Fig. 3). As ALOX12 is released, oxidized membrane PUFAs produce lipid peroxides and initiate ferroptosis [58].

ALOXE3 was reported to induce ferroptosis similar to ALOX12 in glioblastoma (GBM) cells [59]. Under normal conditions, SLC7A11 also sequesters ALOXE3 from its substrate, 12-hydroperoxyeicosatetraenoic acids (a catalytic product of ALOX12). In response to stress, p53 inhibits SLC7A11, resulting in release of ALOXE3 and decreased production and secretion of 12-hydroperoxyeicosatetraenoic acids, followed by induction of ferroptosis. Notably, the p53/SLC7A11/ALOX12 and p53/SLC7A11/ALOXE3 axes independently and cooperatively regulate ferroptosis and participate in tumor suppression, both of which are independent of GSH and GPX4. Hence, the two axes may be a novel way to induce ferroptosis beside the classic ferroptosis model.

#### P53 and iPLA2β

In addition, there is a novel antiferroptosis mechanism that is independent of GPX4 and FSP1. After cleaving the peroxidized PUFAs of membrane PLs, free radicals are released and cleared by various cellular antioxidant systems to solve the problem of PL peroxidation [60]. The core executor of this mechanism is iPLA2β (also called phospholipase A2 group VI, PLA2G6), a calcium-independent phospholipase. IPLA2β is also a target of p53, and can eliminate acyl tails from lipids and release oxidized fatty acids, which are further detoxified by antioxidants in the cytoplasm, leading to negative regulation of ferroptosis [60, 61]. The p53-GPX4 axis eliminates lipid peroxides and stabilizes the internal environment, while the p53-iPLA2β axis solves the abnormal radical disturbance. However, the reactions are complex and context dependent. When lipid peroxidation damage is low and can be repaired, p53 transactivates iPLA2β to restrain ferroptosis. However, if the damage persists or is too severe, ferroptosis induced by p53 will occur with the removal of damaged cells [57]. In particular, p53 and iPLA2β are tightly associated with tumors and neurodegenerative diseases, especially Parkinson’s disease. The p53-iPLA2β axis and other antiferroptosis pathways work together to protect against cell death due to iron overload and ensure the proper physiological benefits of ferroptosis.

### P53 bidirectionally regulates ferroptosis

P53 positively or negatively regulates ferroptosis in different cell types and in response to different stress factors through several independent signaling pathways. As mentioned earlier, p53 promotes ferroptosis mainly by promoting activity of SLC25A28 and expression of FDXR, inhibiting transcription of SLC7A11, promoting expression of GLS2, and increasing the levels of SAT1-ALOX15. This promotion of ferroptosis helps the body to eliminate tumor cells and abnormal cells.

P53 can reduce the sensitivity of cells to ferroptosis and promote normal cell survival, especially when the injury or stress is mild. Positive or negative regulation of ferroptosis by p53 also depends on the cell type. Xie et al. found that p53 wild-type colorectal cancer (CRC) cells were insensitive to erastin, but the sensitivity of these cells to erastin was restored upon inhibition of p53 activity by gene knockout or drugs [56]. Further mechanistic research found that p53 has differential effects on SLC7A11 expression in CRC and non-CRC cells. Inhibition of p53 increased SLC7A11 mRNA and protein expression in U2OS and MCF7 cells. By contrast, inhibition of p53 reduced SLC7A11 expression in human CRC cells. This research revealed that p53 can promote transport of DPP4 into the nucleus and thus reduce the impact of DPP4 on NOX1, thereby reducing the levels of lipid ROS and ferroptosis.

P21 (also known as cyclin-dependent kinase inhibitor 1 A, CDKN1A) is also a target of p53. It mediates the function of p53 by inducing cell cycle arrest and senescence in response to stress signals [62, 63]. Meanwhile, p21 can be induced by p53 to increase production of GSH, which further promotes the function of GPX4, leading to increased elimination of toxic lipids and ROS to inhibit ferroptosis [64].

## Translational research

Translational research of ferroptosis regulation by p53 mainly focuses on tumors and ischemia–reperfusion injury. Preclinical studies have shown that upregulation of ferroptosis can induce tumor regression, while downregulation of ferroptosis reduces ischemia–reperfusion injury of heart, brain, and lung tissues. Thus, there are potential clinical applications to target p53 and ferroptosis.

Regulation of ferroptosis by p53 not only plays an important role in tumor suppression but also enhances sensitivity of tumor cells to radiotherapy, and even has predictive value for antitumor efficacy and prognosis. D13, a novel compound isolated and extracted from the natural saponin Albiziabioside A, triggers apoptosis and ferroptosis of human colon cancer HCT116 cells by activating p53 through the mitochondrial pathway [65, 66]. It has strong inhibitory activity against multidrug-resistant cancer cells. Interestingly, this compound exhibits highly selective inhibition of tumors without eliciting toxic effects on normal organs in vivo. Other ferroptosis inducers have yielded promising data in research of nonsmall cell lung cancer, ovarian cancer, castration-resistant prostate cancer, triple negative breast cancer, colon cancer and NK/T cell lymphoma [67–72]. Interfering with tumor metabolism to induce ferroptosis is a novel antitumor treatment strategy. In addition, treatment with a combination of ferroptosis inducers and radiotherapy has yielded satisfactory results. Induction of ferroptosis by radiotherapy is related to activation of p53 and often suggests a better prognosis. However, expression of SLC7A11 induced by radiotherapy plays a key role in tumor progression and radio-resistance, especially in tumors with p53 mutations or deficiency. Notably, ferroptosis inducers can overcome tumor escape mediated by SLC7A11 and restore the sensitivity of p53 mutant tumors to radiotherapy [73]. In the future, treatment with a combination of molecular targeted drugs, chemotherapy, and immune checkpoint inhibitors together with ferroptosis inducers may yield more promising data. In addition, ferroptosis has prognostic value for certain cancers. A prognostic model including prognosis-related differentially expressed ferroptosis-related genes in head and neck squamous cell carcinoma was built to guide precise and individualized treatment [74]. The prognostic score of this model is closely related to p53 mutation, treatment with six cytotoxic drugs, and expression of immune checkpoint inhibitor-related genes. This ferroptosis-related gene model can not only predict the therapeutic effect in patients with head and neck tumors but also determine the prognosis of these patients. Translational studies of ferroptosis regulation by p53 will lead to increasingly accurate models for tumor treatment, efficacy prediction, and prognosis judgment.

Studies of ferroptosis regulation by p53 have also yielded promising results in alleviating ischemia–reperfusion injury. Acute lung injury is a life-threatening disease with high mortality. Interestingly, animal studies have shown that ferroptosis is involved in acute lung injury triggered by ischemia–reperfusion injury [75]. Inhibition of p53 transcriptional activity can protect normal lung tissue from acute injury induced by ischemia–reperfusion injury via the nuclear factor-like 2 signaling pathway. This provides a potential therapeutic option for acute lung injury caused by ischemia–reperfusion. Acute myocardial infarction (AMI) accounts for a considerable proportion of fatalities. Myocardial reperfusion by thrombolysis or using a stent is the basic therapeutic strategy for AMI. However, ischemia–reperfusion injury cannot be ignored. Recent studies in animal models have shown that overexpression of USP22, SIRT1, and SLC7A11 can inhibit p53 transcriptional activity and thereby ferroptosis, reducing infarct size and improving cardiac function [76]. This provides a new strategy to overcome ischemia–reperfusion injury after AMI treatment. A common site of ischemia–reperfusion injury is the central nervous system. Cerebral ischemia is a common cause of death of hippocampal neurons, stroke, and fatality. Modulation of ferroptosis by SLC7A11 and p53 affects the role of hippocampal neurons [77]. Alteration of the ferritin level can reduce ferroptosis mediated by p53 and SLC7A11. This may be a novel mechanism underlying the possible neuroprotective effect of ferritin on free radical injury and has potential clinical value. In another study, increasing expression of SLC7A11 and GPX4 and inhibiting activation of p53 could reduce early brain injury associated with ferroptosis [78]. Inhibiting ferroptosis by suppressing p53 may be a novel therapeutic strategy for early brain injury. Thus, blocking ferroptosis regulated by p53 activation may become a new strategy for treatment of ischemic diseases.

## Conclusion

Regulation of ferroptosis by p53 is very complex and delicate. Different cell types, different stress factors, and even different intensities of the same stress factor may trigger different p53 signaling pathways and lead to different cell fates. Control of ferroptosis by p53 through canonical and noncanonical pathways is context-dependent. Many details of the mechanisms still need to be clarified. Further translational research will help to elucidate these mechanisms and master regulation of ferroptosis by p53 to tackle tumors and other diseases.
